# Conjugated linoleic acids as functional food: an insight into their health benefits

**DOI:** 10.1186/1743-7075-6-36

**Published:** 2009-09-18

**Authors:** Sailas Benjamin, Friedrich Spener

**Affiliations:** 1 Department of Biochemistry, University of Münster, 48149 Münster, Germany; 2 Biotechnology Division, Department of Botany, University of Calicut, Kerala - 673 635, India; 3 Department of Molecular Biosciences, University of Graz, Heinrichstrasse 31, 8010 Graz, Austria

## Abstract

This review evaluates the health benefits of the functional food, conjugated linoleic acids (CLA) - a heterogeneous group of positional and geometric isomers of linoleic acid predominantly found in milk, milk products, meat and meat products of ruminants. During the past couple of decades, hundreds of reports - principally based on *in vitro*, microbial, animal, and of late clinical trials on humans - have been accumulating with varying biological activities of CLA isomers. These studies highlight that CLA, apart form the classical nuclear transcription factors-mediated mechanism of action, appear to exhibit a number of inter-dependent molecular signalling pathways accounting for their reported health benefits. Such benefits relate to anti-obesitic, anti-carcinogenic, anti-atherogenic, anti-diabetagenic, immunomodulatory, apoptotic and osteosynthetic effects. On the other hand, negative effects of CLA have been reported such as fatty liver and spleen, induction of colon carcinogenesis and hyperproinsulinaemia. As far as human consumption is concerned, a definite conclusion for CLA safety has not been reached yet. Parameters such as administration of the type of CLA isomer and/or their combination with other polyunsaturated fatty acids, mode of administration (*eg*., as free fatty acid or its triglyceride form, liquid or solid), daily dose and duration of consumption, gender, age, or ethnic and geographical backgrounds remain to be determined. Yet, it appears from trials so far conducted that CLA are functional food having prevailing beneficial health effects for humans.

## Introduction

**Figure 1:**
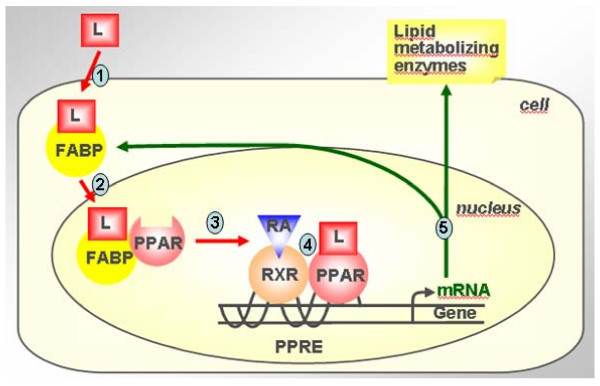
**Proposed CLA-mediated signal transduction**. The dietary lipid nutrient L (L = here CLA) crossing the cell membrane with the help of specific membrane-bound fatty acid transporters and binds to tissue-specific fatty acid binding protein (FABP) in the cytosol; 2. The L/FABP complex enters in to the nucleoplasm, where L is transferred to the specific peroxisome proliferators activated receptor (PPAR) subtype; 3. The L/PPAR complex heterodimerises with retinoic acid (RA)/retinoic acid receptor (RXR) subtype; 4. This heterodimer binds to the peroxisome proliferator responsive element (PPRE) on the target gene; and 5. Specific gene expression occurs, whose products act intra- or extracellularly to elicit a host of various biological functions

Conjugated linoleic acids (CLA) represent a heterogeneous group of positional and geometric isomers of linoleic acid, which are predominantly found in milk, milk products, meat and meat products of ruminants [1,2]. Like neutraceuticals, being minor lipids with supposed functional food status, CLA are getting momentum in alleviating major killer diseases such as cancer, atherosclerosis, and diabetes in humans [3-7]. Despite hundreds of reports, it seems difficult to deduce a common mechanism or molecular basis for the CLA action in *in vivo *conditions, pertaining to reported health benefits [8]. Moreover, contrasting functionalities of CLA isomers make this attempt more difficult [9,10]. Yet, the CLA action at molecular level lies predominantly on the classical CLA-mediated activation of peroxisome proliferator-activated receptors (PPARs) and subsequent "switching on and/or off" of the target genes to elicit a host of biochemical pathways [11]. As shown in Figure 1, during gene regulation, the well characterised PPARs (α, β or γ subtypes) bind to the peroxisome proliferator responsive element (PPRE) on the nuclear DNA as heterodimers with one of the α, β or γ subtypes of the retinoic acid receptor (RXR), which in turn, needs to be activated by *cis*-9-retinoic acid to effect target gene transcription [12,13]. Moderate binding efficiencies of CLA isomers to PPAR subtypes [2,14,15] and potentials for regulating the target genes as shown by transactivation [2,16,17] and expression [18-21] studies substantiate the above molecular mechanism of action.

While PPARs are involved in the regulation of metabolic, immune and inflammatory processes, only a very few studies were conducted to integrate receptor-dependent processes [22]. Due to the therapeutic significance of selective agonists, the scientific understanding of PPARs biology derives primarily from experiments that utilized synthetic ligands to receptors [22]. In addition to the PPAR-mediated actions, various inter-connected downstream molecular mechanisms exist in *in vivo *environment accounting for the reported biological functions of CLA. Moreover, poly unsaturated fatty acids (PUFAs) like CLA and their various metabolites can act at the level of the nucleus, in conjunction with other nuclear receptors and transcription factors, to affect the transcription of a variety of genes. They include hepatocyte nuclear factor (HNF)-4α, and liver × receptor, nuclear factor-κB (NFκB) and the transcription factors sterol-regulatory element binding protein (SREBP)[23]. Mitogen-activated protein kinase/extracellular signal-related kinase (MEK/ERK) signalling through the autocrine/paracrine actions of interleukins-6 and 8 opens up another important route for adipocyte delipidation by CLA [24]. Modulatory effects of CLAs [25] juxtaposed to PPAR-mediated gene expression to effect novel molecular signalling pathways, which is largely mediated though leptins [26,27], adiponectin [28], eicosanoids [29], vitamins [30], immunoglobulins [31,32], and thus altering membrane protein characteristics [33] are also being elucidated. However, objective evidences for these pathways are inconclusive, which demand elaborate studies, especially the involvement of hormones.

In this context, it is appropriate to have an overview into the fundamental basis for major health benefits attributed to CLA, *viz*., anti-obesitic, anti-carcinogenic, anti-atherogenic, anti-diabetagenic, immunomodulatory, apoptotic and osteosynthetic effects. Yet, they are coupled with negative effects like fatty liver and spleen, induction of colon carcinogenesis and hyperproinsulinaemia [34-36].

## CLA isomers

**Figure 2:**
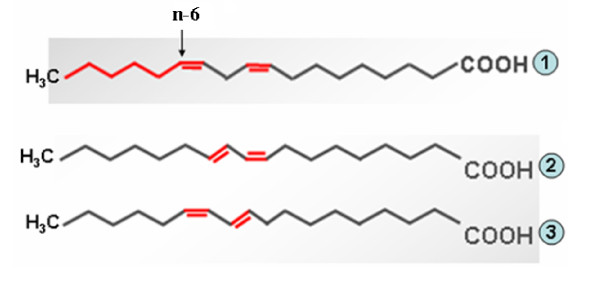
**Structure of linoleic acid and its major CLA derivatives**. 1. Linoleic acid (typical n-6 PUFA); 2. *cis*-9, *trans*-11-octadecadienoic acid (9-CLA, in fact an n-7 fatty acid); 3. *trans*-10, *cis*-12-octadecadienoic acid (10-CLA).

Positive health effects attributed to CLA are mainly based on cell culture models and animal studies with comparatively less scientific evidences from direct studies on humans [4]. Similarly, the molecular mechanism underlying their effects in anti-obesity, anti-carcinogenic, anti-diabetagenic, anti-artherogenic, immunomodulatory, and even few negative effects are yet to be unveiled fully. Natural products, specifically dairy fats, reportedly contain over 25 CLA isomers and by chemical synthesis or modification of the existing ones, novel isomers are being added to this list [1,37]. Hence, the biological activities attributed to CLAs have to be confirmed, if such attributes are due to a single isomer or of the mixture. Being the predominant isomers, *cis-9, trans-11*-CLA (9-CLA, the rumenic acid) and *trans-10, cis-12*-CLA (10-CLA) are the primary focus of most of the studies evaluating the biological activities of CLA [1,2], which are primarily derived from linoleic acid, a typical n-6 fatty acid (Figure 2). Hence, very often, all the CLA isomers are erroneously termed as n-6 fatty acids, yet for instance, 9-CLA is an n-7 fatty acid. Interest in these isomers was stimulated by results obtained in studies using commercially available CLA, which is a mixture of approaximately equal amount (~40% each) of these two isomers [38]. Currently, the trend is in using a single isomer (mostly 9- or 10-CLA) with purity above 90% [2,39]. Apart form the specific anti-obesitic and hypocholesterolaemic effects of 10-CLA [40], most of the rodent (rat and mice) studies suggest that a CLA mixture (9- and 10-CLAs) could be more beneficial for the management of insulin resistance [38]. Such studies in obese humans, however, show that CLA do not affect glucose metabolism or insulin sensitivity [41]. Thus, the biological functions reported for CLA cannot be explained by a single biochemical mechanism or of the activity of a single isomer. It appears that 9-CLA elicit more general biological events than 10-CLA [9,19,21,33,42,43]. Dietary supplements of CLA as free fatty acids [44] or its triglyceride form [45] is also a matter of debate, which demands more evidences.

Among the naturally occurring CLAs, predominantly the 9-CLA but in small amounts also 10-CLA are synthesised in the rumen of cattle, deer, sheep and goat by biotransformation of forage-derived unsaturated fatty acids such as oleic acid and linoleic acid [2]. The *trans*-Δ11-vaccenic acid (t-VA), produced as a rumen biohydrogenation intermediate from both linoleic acid and α-linolenic acid provides amajor alternate route for 9-CLA biosynthesis in mammalian cells, including humans, *via *Δ^9^desaturation by stearoyl-CoA desaturase (SCD) [2]. Strategies to enhance milk fat CLA involve increasing rumen outflow of *t*-VA and increasing SCD activity, and through these, several-fold increases in the content of CLA in milk fat can be routinely achieved through the use of diet formulation and nutritional management of dairy cows [46]. CLA represent minor lipid nutrients in feed and food, in particular in dairy beef products. Milk contains over 20 isomers of CLA but the predominant one is 9-CLA (75-90% of total CLA) [46]. Schmid *et al*. reviewed the intramuscular CLA concentrations in meat and meat products originating from different animal species, factors influencing these concentrations, the estimated human daily intakes and the percentage of CLA provided by meat and meat products [47]. Our group also demonstrated the endogenous synthesis of 9-CLA from precursor *t*-VA in humans [48].

## Anti-obesity

Considering anti-obesitic and hypolipidemic effects, it is possible to modify body composition by supplementing CLA to the diet. Major biochemical actions of CLA associated with anti-obesity are summarised in Table 1[49-54]. Dietary supplementation of CLA has been effective in reducing the percentage of body fat and increasing the percentage of body protein [41,55]. Studies with rodents, pigs and cattle show that effects of CLA on body composition appear to be due in part to reduced fat deposition and increased lipolysis in adipocytes [56], possibly coupled with enhanced fatty acid oxidation in both muscle cells and adipocytes as in rat [26]. Jiang *et al*. [8], in their cross-species (cattle breeds: Wagyu × Limousin) study, found SCD-1 gene to be a critical player in skeletal muscle fat metabolism resulted in high amount of monounsaturated fatty acids (MUFA) and CLA content, but with low amount of saturated fatty acids. CLA mixture (9- and 10-CLA) showed to suppress the SCD activity in porcine subcutaneous adipose tissue [57].

**Table 1:** Major biochemical actions of CLA on lipid metabolism.

**Biochemical action**	**Experimental evidence**	**Ref**
Preadipocyte proliferation	Inhibited proliferation	[49]
Preadipocyte differentiation	Human preadipocytes do not differentiate in the absence of a PPARγ ligand like CLA	[25]
Fatty acid oxidation	Carnitine palmitoyltransferase activity increased by dietary CLA	[28,50]
Adipose tissue lipid synthesis	Inhibition of de novo lipogenesis through down regulation of acetyl-CoA carboxylase and fatty acid synthase	[51]
Lipolysis	Increased lipolysis and decreased fat	[52]
Energy expenditure	Increased oxygen consumption and energy expenditure by 10-CLA	[44]
Stearoyl-CoA desaturase	Inhibition at protein or activity level, by post-translational modification	[15]
Plasma leptin	Decrease in serum leptin, a hormone regulating fat level	[26,53]
Apotopsis	Induce apoptosis in adipocytes	[49]
Tumor necrosis factor-α	Increased expression of TNFα and low fat	[54]

Our group already demonstrated that dietary supplementation with *trans*-11- and *trans*-12-18: 1 MUFA increases 9-CLA in human peripheral blood mononuclear cells lipids (from 0.07 to 0.16%) without effects on biomarkers of immune function and inflammation [48]. In the same intervention study we found that higher body fat accumulation was significantly associated with higher SCD-1 isoform expression in control women, who had 25% more body fat mass than men [58]. Interestingly, after 42 days of intervention, SCD-1 and glucose transporter genes (GLUT 1 and 4) were 10 fold down-regulated in women, but no significant change in men; this effect can be attributed to the endogenously synthesised 9-CLA [48,58,59]. In addition, we also found that stored fats had a strong association with gender-based higher synthesis of hormones such as leptin. Female subjects possessed significantly higher plasma leptin and lower adiponectin concentrations than their male counterparts [59]. This clearly indicates hormonal-related gene regulation [60]. As SCD-1 is the rate-limiting enzyme in the synthesis of MUFA, it becomes the critical control point regulating hepatic lipogenesis and lipid oxidation [61]. The inhibitory effects of 10-CLA on SCD-1 have been consistent, regardless of the model or species [62]. Treatment with mixed isomers, or more specifically 10-CLA, decreases either the activity or abundance of SCD-1 in a human breast cancer cells, human hepatocytes and murine adipocytes [62].

Paton and Ntambi demonstrated SCD-l to be a key enzyme in regulating hepatic lipogenesis and lipid oxidation; therapeutic manipulation of SCD-1 can be of benefit in treatment of obesity and metabolic syndrome [63]. As several manifestations of the metabolic syndrome and type 2 diabetes mellitus are associated with alterations in intracellular lipid partitioning, SCD1 has emerged as a therapeutic target in the treatment of obesity and the metabolic syndrome, according to Dobrzyn and Ntambi [61].

Decreased adipocyte lipid synthesis is observed in a number of studies. *In vivo *and *in vitro *evidences to date suggest that there exist a multiple mechanism for the CLA-mediated fat reduction, in addition to the PPAR-mediated primary mechanism for CLA uptake. It seems that CLA might act as an agonistic or antagonistic ligand for PPARγ to control preadipocites [64]. Interestingly, 9- and 10-CLA isomers equally reduced lipid deposition in porcine preadipocytes with a preferential effect of 10-CLA and no isomer distinction in human preadipocytes [64]. Moreover, CLA might facilitate decreased energy or food intake and increased energy expenditure, decreased preadipocyte differentiation and proliferation, decreased lipogenesis, and increased lipolysis and fat oxidation [5]. Decreased plasma leptin, increased adipocyte apoptosis and increased plasma tumour necrosis factor-α (TNFα) are other possible routes [64]. The fundamental basis for the generalised mechanism lies in the modulation of the transcription factor PPARγ or adipocyte determination and differentiation-dependent factor 1, which would elicit a cascade of multiple metabolic pathways including inhibition of SCD-1 [64,65]. Delipidation by mitogen-activated protein kinase or extracellular signal-related kinase signalling is evidenced by the anti-proliferative activity of CLA in MCF-7 cells [66]. Additional to major mediator effect of TNFα in inflammatory responses, the correlation between its increased expression and decrease in fat deposition [48,67] may be due to increased lipolysis [26] and decreased lipoprotein lipase activities [68].

Studies made on animals and humans show that the consumption of CLA leads to loss of fat and total body weight, reduces the plasma concentrations of total and low density lipoprotein (LDL)-cholesterol, and has an anti-inflammatory effect (Table 1). Gene-by-diet interactions play an important role in the prevention of several diseases. To elaborate this concept, we investigated the changes in gene expression in monocytes upon intervention with two *trans *fatty acids (*trans*-11 18:1 and *trans*-12 18:1) and endogenous synthesis of CLA from *t*-11 18:1 (*t*-VA) as precursor in humans [59]. Expression patterns of 20 candidate genes involved in glucose and lipid metabolism have been assessed, which were chosen on the basis of the interaction of their proteins in fatty acid signalling and are partly regulated by PPAR and have a PPRE in their promoter. Interestingly, the expression patterns revealed prominent gender-based differences in gene expression [59].

Ntambi's group demonstrated that loss of SCD-1 function protects mice from developing obesity [63]. This is likely due to decreased synthesis of long-chain MUFAs such as oleate, a preferred substrate for triglyceride synthesis. The inhibitory effects of 10-CLA on SCD-1 have been consistent, regardless of the model or species. Treatment with mixed isomers, or more specifically 10-CLA, decreases either the activity or abundance of SCD-1 in a human breast cancer cells, human hepatocytes and murine adipocytes [63]. In humans, although it is indicated that 10-CLA is the antiadipogenic isomer, the effects of CLA on fat deposition are less significant and more equivocal as compared to results observed in animals [69]. A need exists to establish whether interactions exist between dietary CLA supplementation and factors such as energy intake, dietary fatty acid composition, especially PUFA in humans. Proposed antiobesity mechanisms of CLA include decreased energy/food intake and increased energy expenditure, decreased preadipocyte differentiation and proliferation, decreased lipogenesis, and increased lipolysis and fat oxidation [69]. Brown and McIntosh [62] proposed that 10-CLA exerts its partial inhibition of adipocyte differentiation by reducing the expression of PPAR γ and its downstream targets that are critical for fatty acid (i.e., ACBP, A-FABP, LPL, perilipin) and glucose metabolism (*i.e*., GLUT4, ACC, SCD-1). 10-CLA decreased the triglyceride content of newly differentiated human adipocytes from stromal vascular cells by inducing MEK/ERK signaling through the autocrine/paracrine actions of interleukins-6 and 8 [24]. The 10-CLA may activate IL-6 and IL-8 gene expression and secretion, which is dependent on an autocrine activation of MEK/ERK signalling in nonadipocyte stromal vascular cells, which in turn, through paracrine actions impacts MEK/ERK signalling in newly differentiated adipocytes, leading to insulin resistance and delipidation [24].

Van Erk *et al*. [70] observed in humans that upon consumption of a specially designed spread with higher levels of medium-chain triglycerides and PUFA (18:2 and 18:3), CLA favoured higher expression of genes related to lipid metabolism and lower activity of inflammatory genes. A short-term diet change elicited the activity of genes that play a role in inflammatory processes in fat tissue of people who are overweight [70]. This technique is an example of nutrigenomics research, and it allows to investigate the effect of complex mixtures of functional food ingredients on fat tissues. Such findings show that genes in fat tissue are sensitive to diet changes. The result also strengthens the hypothesis that fat tissue is actively involved in the development of obesity-related disease. Nutrigenomics defines how food and ingested nutrients influence the genome (personalised nutrition) [70]. Though the above studies throw some light into the potential benefits of CLA in humans, additional studies are required to clearly define optimal level of CLA intake, short and long term effects and side effects (if any) of each individual CLA isomer in order to determine its safety and efficacy [71]. Therefore, long-term randomized clinical trials, controlled with placebo, need to be made in large samples of patients to evaluate the efficacy and safety of CLA isomers before its indiscriminate use in human beings can be recommended [5]. Moreover, such trials have to be replicated in other labs too, preferably in different continents and races with varying food styles.

## Anti-carcinogenesis

CLA inhibits cancer by blocking the growth and metastatic spread of tumours. CLA is fast acting, and begins to inhibit both malignant and benign tumours almost immediately [15]. 10-CLA seems to work preferentially through modulation of apoptosis and cell cycle control, while 9-CLA isomer affects arachidonic acid metabolism [42]. Kritchevsky [72] reviewed the inhibitory effects of CLA on chemically-induced skin, stomach, mammary or colon tumours in mice and rats. *In vitro *studies in murine myeloid leukaemia (WEHI-3B JCS) [73] and human colorectal (HT-29, MIP-101) and prostate (PC-3) colorectal [74] cells, as well as *in vivo *human studies on breast [75,76] and prostate [42] cancers showed CLA's best antiproliferative effects. Cellular mechanisms of modulation of carcinogenesis by CLA are numerous and complex. It may be *via *reduction in cell proliferation [77,78], lipid oxidation [5], vitamin A [79] and prostaglandin (PG) [80,81] metabolisms. It is also possible that CLA may interfere with cell transformation through signal transduction [82]. Furthermore, the anticarcinogenic properties of CLA are, at least partially, attributed to thier ability to interrupt the n-6 PUFA metabolic pathway for the biosynthesis of eicosanoids, including PGs [80,81]. Altered phospholipid-associated fatty acid metabolism and eicosanoid (20-carbon derivatives *viz*, PGs, thromboxanes, leukotrienes, hydroxyeicosatetraenoic acids) formation are yet other thrust areas of active research. Eicosanoids modulate cell proliferation, inflammation, local and systemic immunity, platelet aggression and tissue diffrentiation. Free CLAs could compete with other fatty acids to be incorporated in the phospholipids and modifies subsequent eicosanoid production. Dietary CLA reduce PG-E2 [83] and others (PGF_2α_, leukotriene-B_4_, leukotriene-C_4_) derived from arachidonic acid metabolism [29,84,85]. Other possible route for the CLA mediated reduction of arachidonate-derived eicosanoids is through inhibition of cyclooxygenase (COX) 1 and 2. This include pathways for arachidonic acid metabolism [42], apoptosis (bcl-2) and cell cycle control (p21(WAF/Cip1)) [42,43].

## Cell cycle and apoptosis

CLAs could alter growth of neoplastic cells by influencing cell replication, interfering with components of cell cycle, or increasing cell death by promoting necrosis or/and apoptosis. Necrosis generally result from insult or toxicity reaction and triggers inflammation, whereas apoptosis is a distinct energy requiring process of programmed cell death, characterised by DNA fragmentation, chromosome condensation, nuclear fragmentation, formation of apoptotic bodies, and inversion of phosphatidylserine in the plasma membrane [86]. It is expected that CLA could reduce cell proliferation by blocking DNA synthesis [87] and cell cycle proteins [42,88] that regulate this process, and that CLA may support elevated apoptosis primarily by suppressing the expression of antiapoptotic bcl-2 gene [29]. With different cell lines, CLA was able to increase the IL-2 and IFN-γ *via *modulation of protein kinase activity and production of oxidant species, which significantly inhibited proliferation [89], and it appears to be the function of relative content of specific isomers and their ability to elicit a p53 response that leads to cell growth arrest by inhibiting the expression of factors required for G1 to S-phase transition including cyclins D1 and E [90], or by the inhibition of the insulin-like growth factor-I receptor signalling pathway [87].

## Anti-atherosclerosis 

Atherosclerosis is a progressive disease of medium and large arteries by the accumulation of lipids in the inflammatory cells (foam-cell formation), cellular proliferation, platelet adherence and aggression, and calcium deposition [91,92]. CLA are a potent anti-atherogenic dietary fatty acid in animal models of atherosclerosis by activating PPARs [93,94]. One would expect (a) decrease in atherogenic lipoprotein plasma levels such as very low, LDL-cholesterols and increase in anti-atherogenic high density lipoprotein cholesterol (HDL) through increases in apo A-I and apo A-II synthesis; (b) overexpression in HDL receptors capable of increasing cellular cholesterol efflux; and (c) decrease in vascular inflammation by repressing nuclear NF_*k*_B and apo A-I transcriptional activity and they would reduce thrombosis risk by inhibiting tissue factor and fibrinogen synthesis [59,95]. Despite a few studies [5,40,43,96], no conclusive evidences involving CLA isomers are available to substantiate the above signalling pathways proposed for drugs. Interestingly, ratios of the LDL cholesterol to HDL cholesterol and total cholesterol to HDL cholesterol were significantly reduced in CLA-fed rabbits with less atherosclerosis [36,62].

## Anti-diabetes

Diabetes can be caused by too little insulin (type I), resistance to insulin (type II), or by both. Supplementing the diet with CLA may lead to better disease management in diabetics, especially type II. Many studies strongly suggest that the 10-CLA isomer may be the bioactive isomer of CLA to influence the body weight changes observed in subjects with type II diabetes, reviewed by Belury *et al*. [97]. Brown *et al*. observed that during delipidation process, 10-CLA exerts a cascade of molecular actions by down regulating the expression of PPAR γ and its downstream targets that are critical for fatty acid and glucose metabolism, which eventually inhibits glucose and fatty acid uptake and metabolism. [24].

Studies investigating the mechanisms by which CLA operates at the cellular level show that the primary targets for CLA are members of the nuclear receptor family, particularly the lipostat transcription factors; *viz *PPARα, PPARγ, SREBP1c, and LXRα [38,41]. Consequently, the effects of CLA on glucose metabolism are likely secondary effects mediated through factors such as PPARγ coactivator 1[98] that are controlled by these nuclear receptors. Or it could be due to complex mechanisms by the regulation of the expression of genes (like uncoupling proteins) important in the regulation of adipogenesis, glucose and lipid metabolism, and, perhaps, whole-body thermogenesis [99]. Another possible action of CLA in alleviating hyperinsulinemia (in Zucker diabetic fatty rats) is *via *the sensitisation of the adiponectin, a recently discovered hormone secreted by adipocytes that has been reported to enhance insulin sensitivity [100]. Furthermore, determining the ability of CLA isomers to influence glucose and lipid metabolism as well as markers of insulin sensitivity is imperative to understanding the role of CLA, and thus to aid in the management of type II diabetes and other related conditions of insulin resistance [101,102].

## Immunomodulation

*In vitro *studies of the use of immune cells and *in vivo *animal models demonstrate that CLA modulate immune function. However, in contrast to the reports with animal models, CLA feeding to young healthy women did not alter any of the indices of immune status tested, and it suggests that short-term CLA supplementation in healthy volunteers is safe, but it does not have any added benefit to their immune status [103]. Ringseis *et al. *found that CLA inhibit TNFα-induced eicosanoid release from human vascular smooth muscle cells with the interaction of PPARγ [104]. Reports demonstrate that both the active CLA isomers (9- and 10-CLAs) can elicit both the innate and adaptive immune responses [105-107]. These effects lie in the ability of CLAs to modify soluble factors or mediators of immunity such as eicosanoids [108], cytokines [109] and immunoglobulin production [104,110]. Albers *et al. *[105] investigated the effects of two different mixtures of 9- and 10-CLA glycerides [in the ratio 50:50 (1.7 g) or 80:20 (1.6 g) per day, repectively administered for 12 weeks] on human immune function. They found that 50:50 ratio beneficially enhanced the protective antibody levels to hepatitis B. CLA may alter eicosanoid signalling *via *TNF-α, and thus affecting a range of biological functions including antigen presentation [19]. This would cause changes in the membrane characteristics and changes in the activity of membrane proteins that serve as ion channels, transporters, receptors, signal transducers or enzymes [86]. The alternative hypothesis is rooted in the fundamental PPAR-mediated signalling, *via *expression of target genes involved in immune function [19]. CLA can also protect against tissue breakdown due to immune stimulation during periods of severe illness [111,112]. This effect may be due to the modulation of NF_*k*_B for negatively regulating the lipopolysaccharide-induced inflammatory responses [108], or against the catabolism and inflammatory effects induced by cytokines, especially TNFα [19]. The immunomodulatory effects of CLA may have application in livestock production as an alternative to the use of feed antibiotics (functional food), or as a means of improving the response to vaccination and conferring disease resistance.

Song *et al. *investigated the effect of dietary CLA supplementation (3 g/day; 50:50 mix of 9- and 10-CLA major isomers) on the immune system and plasma lipids and glucose of healthy human (male and female) volunteers [113]. Interestingly, levels of plasma IgA and IgM were increased with decreased plasma IgE levels. CLA supplementation also decreased the levels of the proinflammatory cytokines, TNF-α and IL-1β, but increased the levels of the anti-inflammatory cytokine, IL-10. In addition to these effects, delayed type of hypersensitivity response was decreased during and after CLA supplementation, coupled with no significant changes for plasma glucose, lipids, lymphocyte morphology. Very recently, Kwak *et al*. observed in obese pre-menopausal Korean females that CLA (9- and 10-CLA mixture) supplementation modulated to the increased release of markers (C-reactive protein, IL10, IgM) related with inflammation and immune function, and this effect was much more subtle than those found in animals and few other clinical studies [114].

## Bone formation

Mixed CLA isomers have been shown to have variable effects on bone formation (ostheosynthesis) and resorption in animals. The variable effects of CLA on bone physiology may be due to the different isomers present in common commercial preparations of CLA, and the effects of the predominant individual isomers (9- and 10-CLAs) are not clear. Dietary CLA inhibits eddosteal bone resorption, increases endocortical bone formation, and modulates the action and expression of COX enzymes, thereby decreasing prostaglandin-dependent bone resorption [115,116]. CLA also enhances calcium absorption from diet, improve bone formation and reduce the rate of bone resorption in adult OVX rats [117]. Since CLA can affect inflammatory cytokines, it is hypothesized that CLA may be a good tool for prevention or reduction of rheumatoid arthritis symptoms in humans [109]. However, under the conditions tested in this double-blind, placebo-controlled trial in adult men, a CLA supplement of mixed isomers did not affect markers of calcium or bone metabolism [109]. Doyle *et al*. [118] found that supplementation with CLA or placebo for 8 weeks had no significant effects on markers of bone formation (serum osteocalcin and bone-specific alkaline phosphatase) or bone resorption (serum C-telopeptide-related fraction of type 1 collagen degradation products, urinary N-telopeptide-related fraction of type 1 collagen degradation products, urinary pyridinoline and deoxypyridinoline), or on serum or urinary calcium levels in healthy adult men. Baseline levels of these biochemical parameters were similar in both groups of subjects. While the placebo had no effect, CLA supplementation resulted in a three-fold increase in 9-CLA isomer in total plasma lipids [118]. Platt *et al. *have found that alkaline phosphatase activity in cell lysates as a marker of early osteoblast differentiation in human osteoblast-like cells [119]. The 9-CLA increased the number and size of mineralized bone nodules from 25 to 100 μM, but the 10-CLA did not show such effect [119]. The increase in mineralized bone nodule formation by 9-CLA was accompanied by a variable increase in alkaline phosphatase activity. These results show that the 9-CLA increases the formation of mineralized bone nodules using bone cells of human origin, and provide evidence for isomer-specific effects of CLA on bone health [119].

## Safety concerns and human scenario

Though only positive health benefits of CLA have been addressed here, some negative impacts of CLA could not be ruled out [120]. Some human CLA supplementation studies have often shown conflicting and less convincing health benefits. The marked variations between studies may reflect the isomer-specific effects of individual CLA isomers, which can often have opposing effects [36]. Main findings from the mice models are increased liver and spleen weight [16,26] and insulin resistance [41,98]. CLA-induced fatty liver hemorrhagic syndrome in birds [121] is yet another facet. A major problem arising from human studies related mainly to the gastrointestinal tract; data indicate that 10-CLA can act as a cancer promoter in colon carcinogenesis, possibly through pathways affecting NF_*k*_B and cyclin D1 [122]. Though 10-CLA profoundly decreased body fat in mice possibly through increased energy expenditure, dietary CLA greatly increased the activity and mRNA levels of various lipogenic enzymes like hepatic Δ^5^- and Δ^6^- desaturases and SREBP-1 in the liver [123]. A large increase in lipogenesis and accumulation of triacylglycerol in the liver after CLA treatment may represent the physiological response of the animal to metabolize excess glucose to fatty acid for storage as triacylglycerols in liver rather than in adipose tissue [123]. Therefore, there is a possibility that the counteraction of CLA-mediated induction of hepatic lipogenesis aggravates glucose intolerance and hyperinsulinemia, despite being potentially effective in preventing fatty liver [123].

In obese men, 10-CLA induces hyperproinsulinemia that is related to impaired insulin sensitivity (hyperinsulinaemic-euglycaemic clamp), independently of changes in insulin concentrations [124]. As hyperproinsulinemia predicts diabetes and cardiovascular diseases, the use of weight-loss supplements containing 10-CLA cautions its indiscriminate consumption. There may be divergent effects of CLA isomers in obese or diabetic subjects compared to the normal-weight or healthy subjects as well as differences determined by gender and/or genetics, *i.e*., single nucleotide polymorphisms in related genes [125]. Moreover, such studies need to be duplicated in other labs giving emphasis to men and women, age groups, ethnic background, or food style. The possible beneficial effects of 10-CLA supplementation in decreasing body fat mass have received a great deal of attention, but potential adverse effects of CLA on the insulin balance have been largely ignored [69]. This is paradoxical, because CLA-mediated hyperinsulinemia has been observed in several studies in mice. CLA-induced insulin resistance may be related to the alterations of plasma leptin levels. Studies have shown that CLA supplementation induced reductions of plasma leptin levels in various animal models [69]. The inconclusive results in human supplementation trials are due to the use of mixed isomers, which may negate one another, resulting in no net change in adiposity; moreover, doses used in human trials were much lower than those used in animal studies [62]. 10-CLA decreases the expression of PPAR γ in adipocytes, which could promote insulin resistance. These paradoxical findings may arise from the use of mixed isomers of CLA or the difference in experimental models used. In any event, isomer-specific dose-titrated clinical studies combined with mechanistic studies in cultures of primary cells should provide the much needed insight on potential human applications for CLA. Within various rodent species and strains, dietary CLA exerts varying potencies; therefore, the differences in species' sensitivities are of great importance when trying to extrapolate rodent data to the human situation [2,15].

Of late, over a hundred clinical studies regarding the efficacy of CLA as functional food are available in literature. A brief survey on the outcome of such studies shows that the overwhelming beneficial effects of CLA impart positive outlook. Table 2[113,125-135] gives some of the notable effects of CLA in humans, as evidenced by clinical studies. It seems that 9- and 10-CLA are having contrasting biological functions, but 10-CLA with more detrimental effects. However a 50:50 ratio of these isomers may give better effect, as effected in immune function [105]. Normal CLA concentration in human body is 0.1% of the total fatty acid composition [136]. Studies reveal that an intake of about 2-3 g per day for 6 to 12 months by an adult would impart optimum biological effects, which would be long lasting too [137]. According to Fernie *et al*. [138] CLA as triacylglycerol is the most suitable form for consumption and that the 9- and 10-CLA isomers are absorbed similarly into chylomicrons.

**Table 2:** Important biological effects of CLA in human subjects.

**Treatment**	**Effect**	**Ref**
CLA with creatine	CLA and creatine as adjuncts increased mictochondrial function by reducing sarcopenia and decreased oxidative stress in older adults	[116]
CLA with ω-3 fatty acids	prevents increased abdominal fat and increases fat-free mass and adiponectin.	[28]
9-CLA	No adverse effect on coronary vascular disease	[126]
9-CLA	Modest anti-inflammatroy effect in allegic subjects	[32]
CLA with γ-oryzanol	Reduced blood pressure and body fat.	[127]
9-and 10-CLA	Enhanced fat oxidation and energy expenditure during sleep	[50]
10-CLA	Anti-lipogenic effect in lactating women's mammary tissue	[128]
CLA	Reduce weight gain induced by psychotropic medication	[129]
CLA	Favourable effect on serum insulin, but no effect on body composition, energy expenditure of apetite.	[101]
CLA	Increased resting metabolic rate, PPAR-γ and hormone-sensitive lipase	[68]
CLA	No effect on glucose metabolism or insulin sensitivity on obese population.	[41]
*Trans *fatty acids (*t*-11/*t*-12, 18:1)	Gender-based gene expression	[59]
CLA	Inhibition on leptin, adiponection - contribute to insulin resistance	[26]
CLA and Calcium	Reduces pregnancy-induced hypertension and decreases the intracellular concentration of ionised free calcium in peripheral blood lymphocytes.	[130]
CLA	Do not beneficially change risk factors for cardiovascular disease or diabetes	[94]
CLA and Vaccenic acid	No effect on blood pressure or arterial elasticity in healthy young men.	[131]
CLA	Affect lipid and carbohydrate metabolism and reduced body weight	[27]
CLA and other PUFA	Possible role in preventing renal carcinoma	[132]
CLA	Reduce colorectal carcinoma	[133]
CLA	Enhanced immunological function	[102,113]
CLA	Enhanced C-reactive protein	[43]
CLA	Suppresses rheumatoid arthritis	[109]
CLA	Lipid peroxidation	[124]
CLA	Not good to treat metabolic syndrome	[134]
CLA	Not associated with breast cancer	[76]
10-CLA	Induces hyperproinsulinaemia, which predicts diabetes and cardiovascular disease	[124]
CLA	Ptotective effect on the risk of metastasis in breast cancer	[75]
10-CLA	Increases oxidative stress and inflammatroy biomarkers in obese men	[135]
CLA	Positive impact on cardioprotective effect.	[89]
CLA	Modulation of risk factor associated with atherosclerosis	[125]

Very recently Kelley *et al. *demonstrated in mice that some adverse effects like insulin resistance and non-alcoholic fatty liver disease attributed to CLA may be due to the deficiency of n-3 PUFA and that such adverse effects can be corrected by a concomitant increase in the intake of α-linolenic acid, an n-3 PUFA and flax seed oil, a rich natural source for this fatty acid [139]. Considering the reported adverse events and safety concerns, Gaullier *at al. *assessed the effects of supplementation of 3.4 g/d CLA (1:1 ratio 9- and 10-CLA in triglyceride form) in 134 humans for 2 years [140]. The data revealed that CLA supplementation for 24 months in healthy, overweight adults was well tolerated as revealed by the decreased body weight and body fat mass, and increased circulating lipoprotein, thrombocytes, and aspartate amino transferase. There was no change in fasting blood glucose. Plasma total cholesterol and LDL cholesterol were reduced, whereas HDL cholesterol and triglycerides were unchanged. The reported adverse effect rate was decreased considerably in the 2-years long study, compared with the initial 12 months of the study [140]. These results indicate that most of the reported adverse effects are related to the short-term studies in humans [140]. Apparently, many of the physiological adverse effects like hyperinsulinemia and fatty liver in mice were ameliorated with the inclusion of increasing amounts of fish oil in the diets, which is a rich source for very long-chain fatty acids [123]. These results indicate that a mixture comprising all n-3, n-6 (CLA) and n-9 fatty acids in an appropriate proportion on humans would be a better answer to avoid the reported adverse effects of CLA.

## Conclusion

The heterogeneity of both *in vitro *and *in vivo *evidences on the efficacy of CLA studies makes it difficult to pin-point whether CLA offer a 100% safe functional food. Obese people are likely to consume more of these minor lipid nutrients, irrespective of their high cost. Although comparatively few human clinical studies exist, it appears to date that CLA are beneficial for human health. More focused world-wide network clinical trials involving probands and patients from all continents are required to arrive at conclusive evidence. Another important aspect is contrasting functionalities of CLA isomers and the fact that a majority of clinical trials use a crude mixture of CLA (predominated by 9- and 10-CLAs). Moreover, the reported negative effects like fatty liver and spleen, induction colon carcinogenesis, are yet to be proved beyond doubt. Furthermore, apart from PPAR-mediated signalling, more conclusive evidences are necessary to unravel other molecular mechanisms and complex signalling pathways triggered by dietary CLA.

Strictly controlled studies as performed in animals or in culture models may not be maintained in clinical trials, however, most of human studies are based on blood, blood cells, milk or biopsy specimens - all these would cause probable variations in the general data generated. Thus, conclusive studies focused on parameters such as type of CLA isomer administered, variables measured, mode of administration (*eg*., as free fatty acid or its triglyceride form, liquid or solid), gender, age, and ethnical background remain to be taken on prior to conclude that CLA is a fool-proof functional food to humans. To this end, a positive result is that recent studies emphasise a combination of CLA with PUFA to be best formula to ameliorate the adverse effects observed so far.

## Competing interests

The authors declare that they have no competing interests.

## Authors' contributions

Both authors contributed equally to this article, read and approved the final version of the manuscript.
